# 非小细胞肺癌血清标志物PACAP的蛋白质组学筛选和鉴定

**DOI:** 10.3779/j.issn.1009-3419.2012.05.07

**Published:** 2012-05-20

**Authors:** 恒 张, 胜喜 陈, 凌瑾 黄

**Affiliations:** 410008 长沙，中南大学湘雅医院心胸外科 Department of Cardiothoracic Surgery, Xiangya Hospital, Central South University, Changsha 410008, China

**Keywords:** 肺肿瘤, 血清标志物, 半胱天冬酶凋亡前衔接蛋白, Lung neoplasms, Serum marker, Proapoptotic caspase adaptor protein

## Abstract

**背景与目的:**

早诊断早治疗可以提高肺癌的长期生存率，血清标志物可能有助于肺癌的早期诊断。本研究在组织培养的基础上，应用蛋白质组学方法筛选潜在的肺癌血清标志物。

**方法:**

采用差异蛋白质组学技术对肺腺癌和肺组织培养液中的差异蛋白质进行初步鉴定。选取其中的半胱天冬酶凋亡前衔接蛋白（proapoptotic caspase adapter protein, PACAP）做更进一步的研究，用Western blot验证PACAP在培养液上清中的表达情况并检测它在不同类型血清中的表达情况，用ELISA检测不同类型血清标本中PACAP的表达情况。

**结果:**

共得到差异蛋白点19个，鉴定出14个。PACAP在双向电泳中表达差异较大，Western blot显示PACAP在肺癌组织培养液及肺癌患者血清中的表达均明显增高。ELISA显示PACAP在肺腺癌和肺鳞癌患者血清中的表达水平高于在小细胞肺癌、肺良性肿瘤和健康人血清中的水平（*P* < 0.05）。

**结论:**

PACAP是潜在的非小细胞肺癌血清标志物。

## Abstract (en)

**Background and objective:**

Early diagnosis and treatment of lung cancer contribute to a patient's prolonged survival. Proteomics and tissue culture were used to screen potential serum markers of lung cancer.

**Methods:**

The differential proteomic technique and matrix-assisted laser desorption/ionization-time of flight mass spectrometry were used to identify overexpressed proteins from cultured tumor tissues in a conditioned medium. Paired lung tissues in a conditioned medium were used as the control group. Overexpressed proapoptotic caspase adaptor protein (PACAP) was identified using Western blot and enzyme-linked immunosorbent assay (ELISA).

**Results:**

The expression of nineteen protein spots exhibited a twofold increase compared with that in the control group. Fourteen of which were identified, including the distinctly overexpressed PACAP. Western blot analysis revealed a high level of PACAP in the lung adenocarcinoma in both tissue culture media and patients' serum. Overexpressed PACAP was also detected in the serum of non-small cell lung cancer patients via ELISA compared with that in the serum of small cell lung cancer patients, benign lung tumor patients, and healthy individuals (*P* < 0.05).

**Conclusion:**

PACAP is a potential serum marker of non-small cell lung cancer.

肺癌是目前世界上男女性发病率和死亡率均占第一位的恶性肿瘤，肿瘤患者中约25%死于肺癌^[1]^。近年来，世界上肺癌的总体发病率和死亡率呈现增加的趋势。在目前的医疗条件下，早诊断早治疗是提高肺癌长期生存率的非常重要的办法。对于早期肺癌的治疗，现在的方法已经可以取得理想的效果，因此重点即为如何做到早期诊断上面。

对人群特别是高危人群进行筛查是发现肿瘤最常用的手段。在肺癌的筛查上，痰细胞学检查、影像学检查和血清学检查是三个主要的方向^[2]^。痰细胞学检查简便易行，但敏感性和特异性都很低，临床应用价值极为有限。而对于影像学检查，现阶段的筛查意义并不明显^[3]^。

血清学检查是临床上常用的检查方法，易于接受，结果也很客观。试图以血清学检查来达到诊断肺癌的目的基于如下的假设：肺癌细胞与正常细胞在蛋白表达谱、表达量方面均有差异，这些蛋白质可通过细胞分泌、细胞破裂等多种途径进入血液，通过检测肺癌细胞与正常细胞在蛋白表达质和量上的差异、进而筛选肺癌血清标志物是一种高效的肺癌诊断标志物筛选方法。

我们在以往的肺癌细胞蛋白质组学研究中发现，肺癌细胞分泌蛋白是筛选肺癌血清标志物的重要来源^[4, 5]^。然而，肺癌细胞分泌蛋白在在体情况下不一定出现在患者血液中，因此，对肺癌组织中溢出的可溶性蛋白进行研究将为肺癌血清标志物的筛选提供非常有益的线索。本研究试图通过肺癌组织培养，进而对培养液中蛋白成分的通过蛋白质组学方法进行鉴定，从而筛选肺癌血清标志物。

## 1 材料与方法

### 1.1 材料

#### 1.1.1 试剂

鼠尾Ⅰ型胶原（collagen type Ⅰ，杭州生友），特级胎牛血清（Gibco），BrdU（BM0201，武汉博士德），小鼠抗BrdU IgG（ED1100，武汉博士德），即用型免疫组化试剂盒（SA1021，武汉博士德），Bradford蛋白定量试剂盒（Bio-Rad），ACN（色谱纯）（Fisher），IPG strip（pH3-10L, 13 cm; pH4-7L, 13 cm）（Amersham Biosciences），Trypsin Gold（Promega），鼠抗人PACAP（ab67021）（Abcam）。

#### 1.1.2 设备

PROTEAN Ⅱ Xi Cell垂直电泳槽（Bio-Rad），IPGphor等电聚焦仪（Amersham Biosciences），Voyager-DE STR 4307 MALDI-TOF-MS质谱仪（Applied Biosystem），UVS400型真空冷冻干燥仪（Savant speed Vac），Amicon Ultra-4 5KDa离心超滤管（Millipore），Mini-ProTEAN垂直电泳槽（Bio-Rad），Power PAC 3000电泳仪（Bio-Rad）。

### 1.2 病例及标本

#### 1.2.1 组织标本收集

术前病理学确诊的肺腺癌患者6例，男:女为4:2，平均年龄52.2岁，肺癌分期Ⅱ期2例，Ⅲ期4例。在切下的肺组织中采集癌组织和距癌组织至少5 cm的肺组织，共6组。生理盐水清洗干净后，一部分用于组织培养，一部分置于-70 ℃冰箱保存备用。

#### 1.2.2 血清样本

收集于我院明确诊断的患者术前或治疗前的血清标本共144例（患者基本资料见表 1）。标本收集均得到患者许可。抽取动脉血5 mL于促凝管中析出血浆后，上清离心所得即是蛋白质样品。保存于-70 ℃冰箱备用。

**1:** 肺癌患者临床及病理资料 The clinical and pathologic data of lung cancer patients

Group	*n*	Gender (Male/Female)	Mean age (yr)
Squamous cell carcinoma	Stage Ⅰ (*n*=5); stage Ⅱ (*n*=13); stage Ⅲ (*n*=27)	22/23	51+17
Adenocarcinoma	Stage Ⅰ (*n*=3); stage Ⅱ (*n*=18); stage Ⅲ (*n*=22)	20/23	48+10
Small cell lung cancer	8	5/3	45+19
Lung benign tumor	28	17/11	41+23
Health control	20	12/8	48+18

### 1.3 方法

#### 1.3.1 组织培养

培养方法见文献^[6]^。简言之，取同一患者的癌组织和正常肺组织细切后分别置于鼠尾胶原配制成的胶原基质上进行组织培养，每一组均包括腺癌（Xca）、正常肺组织（Xf）（阴性对照组）、DMEM培养液组（空白对照组）。

#### 1.3.2 组织溢出蛋白样品采集

观察肺癌组织及肺组织生长情况，从培养的第4天起至第14天每日更换培养液，更换的培养液先低速离心去除细胞及粗颗粒，再用滤器（0.22 μm）过滤，滤液于-70 ℃的冰箱里保存。使用时，将冻存的滤液先置于4 ℃的冰箱中解冻。再加入离心超滤管中离心。4 mL的样品可浓缩为400 μL。蛋白样品浓度测定采用Brodford法。先分别取1 mg/mL的双乙酰胺：0 μL、2 μL、4 μL、6 μL、8 μL、10 μL、12 μL、14 μL、16 μL、18 μL、20 μL，补实验用缓冲水至终体积100 μL，加入1mL Bradford工作液并震荡混匀，在5 min后测*OD*_595_值。得到标准曲线。再以同样方法取1 μL样品，分别加入99 μL的水，再补以Bradford工作液1 mL，进行样品的OD值测定，再根据得到的标准曲线计算蛋白浓度即是样品本身的浓度。

#### 1.3.3 双向凝胶电泳及图像分析

按之前文献^[7]^描述的方法进行。简言之，分为腺癌组（Xca）和正常肺组织组（Xf），上样量均为1.3 mg。蛋白样品应用IPGphor IEF System进行一相等电聚焦，聚焦结束后，双蒸水洗尽胶条背面的覆盖液，平衡胶条，垂直SDS-PAGE电泳，至溴酚兰指示线到达凝胶底边处停止电泳。取出凝胶玻板，考马斯亮蓝染液染色。Image scanner将染色的凝胶扫描进计算机。借助图像分析软件PDquest进行成组匹配比较。选择表达差异2倍以上的蛋白质点进行质谱分析。

#### 1.3.4 质谱分析及生物信息学检索

选取目标蛋白点，切下后每点加入脱色工作液50 μL，在37 ℃恒温水浴箱中脱色30 min，然后超纯水洗涤胶块，ACN脱水，真空冷冻干燥仪中抽干1 h至胶块完全干燥。每点加200 mmol/L NH_4_HCO_3_溶解的Trypsin（0.04 μg/μL）5 μL，置于37 ℃恒温水浴中酶解18 h，取出离心管，吸出上清，加入20 μL萃取液覆盖胶块，37 ℃水浴30 min萃取，在真空冷冻干燥仪中浓缩至样品体积约为2 μL-5 μL。然后在点样板上的点样孔中先后点上0.5 μL样品和0.5 μL基质液的混合物，置于空气中干燥后用0.1%TFA进行板上脱盐，MALDI-TOF-MS（基质辅助激光解析串联飞行时间质谱仪）分析，Mascot Distiller软件识别所获得的PMF（肽质量指纹谱）图谱单同位素信号峰，将获得的肽片段的质荷比（m/z）数值应用Mascot查询系统对PMF进行检索。搜索数据库为SWISS-PROT和NCBInr蛋白质数据库。

#### 1.3.5 Western blot检测

分别取培养液上清蛋白及血清蛋白按照文献方法进行Western blot检测：取50 μg样品上样，使用Mini-ProTEAN系统进行SDS-PAGE分离和转膜（PVDF），脱脂牛奶封闭后，加入封闭液和一抗（PACAP 1:500），温育2 h后，TBS-T液漂洗膜3次。再加入封闭液和二抗（单抗1:2, 000），摇床上温育1 h，用TBS-T液漂洗膜3次，每次10 min。ECL试剂发光显影，X光片记录。

#### 1.3.6 ELISA检测（双抗体夹心法）

选取144例患者的血清标本，进行ELISA检测。96孔酶标板用缓冲液洗涤后，每孔0.1 mL稀释PACAP抗体包被，4 ℃过夜。次日，弃去孔内溶液，洗涤后，加一定稀释的待检样品血清0.1 mL于上述已包被之反应孔中，37 ℃孵育1 h。洗涤（同时做空白孔、阴性对照孔及阳性对照孔）。加入新鲜稀释的酶标PACAP抗体0.1 mL于各反应孔中。37 ℃孵育1 h，洗涤。加入临时配制的TMB底物溶液0.1 mL于各反应孔中，37 ℃、10 min-30 min。加入2 mol/L硫酸0.05 mL于各反应孔中终止反应。在ELISA检测仪上，于410 nm处，以空白对照孔调零后测各孔*OD*值。

### 1.4 统计学分析

使用SPSS统计分析软件分析各组之间的差异。每组结果都用Mean±SD表示。采用配对样本比较的*Wilcoxon*符号秩和检验或*Kruskal-Wallis* H检验和多个独立样本两两比较的*Nemenyi*检验。*P* < 0.05为差异具有统计学意义。

## 2 结果

### 2.1 凝胶电泳

共得到3个对照组共12份的总蛋白质考染图谱各4张。经PDquest软件分析及人工匹配，每张蛋白数在230个左右。经PDquest软件处理后，共发现差异蛋白质点19个（表达差异2倍以上），差异点范围集中在pI4-pI7和66 kDa-10 kDa之间。图 1是肺腺癌培养液上清蛋白（图 1A）及配对肺组织培养液上清蛋白（图 1B）双向凝胶电泳考染图谱。图 2是差异蛋白第6号点在双向电泳时的差异情况。

**1:**
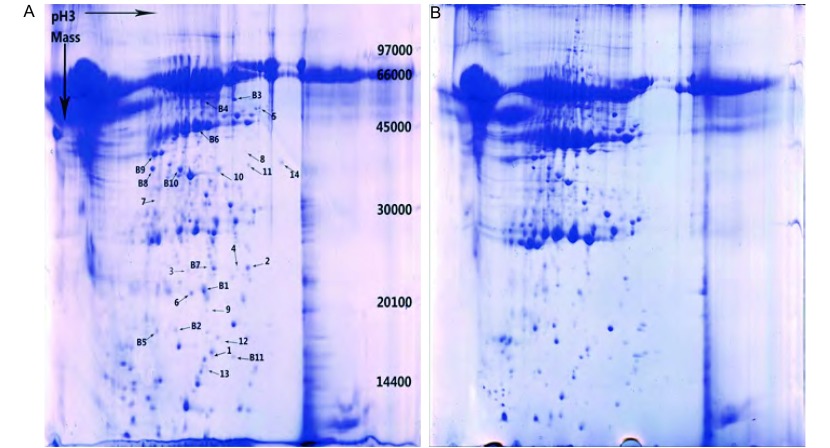
肺腺癌培养液上清蛋白（A）及配对肺组织培养液上清蛋白（B）双向凝胶电泳考染图谱（图中点1-19是人属蛋白质） Coomassie blue dyed 2D Gel electrophoresis of conditioned medium of lung cancer tissue (A) and paired lung tissue (B) (Spot 1-19 are homo sapiens)

**2:**
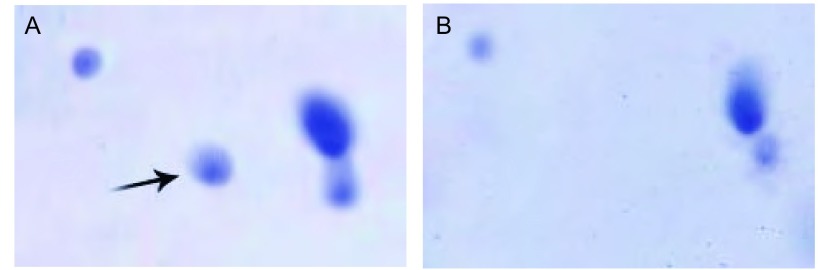
差异蛋白点6号在二维电泳中的表达情况局部图（A代表腺癌组，B代表肺组织组） The partial detail of the sixth differential expressed protein spot in 2D Gel electrophoresis (A stands for lung carcinoma conditioned medium and B stands for that of lung tissue)

### 2.2 差异蛋白质点的生物信息学鉴定

19个差异性蛋白质点经质谱仪分析和数据库搜索，共鉴定14个（表 2）。包括：*α*-烯醇酶（non-neural enolase, NNE）、磷酸丙糖异构酶（triosephosphate isomerase, Tim）、核苷二磷酸激酶A（nucleotide diphosphate kinase A, NDPK A）、儿茶酚O-甲基转移酶2（catechol O-methyltransferase 2, COMT 2）；膜联蛋白A1（Annexin A1, ANXA1）、膜联蛋白A2（Annexin A2, ANXA2）、膜联蛋白A4（Annexin A4, ANXA4）、凝集素-3（Galectin 3）、半胱天冬酶凋亡前衔接蛋白（proapoptotic caspase adapter protein, PACAP）；铜锌超氧化物歧化酶（superoxide dismutase[Cu-Zn], SOD1）、谷胱甘肽-S-转移酶P（glutathione S-transferase P, GSTP1）；转甲状腺素蛋白（transthyretin, TTR）、乙基丙二酸脑病蛋白1（ethylmalonic encephalopathy protein1, ETHE1），PDZ和LIM结构域蛋白1（PDZ and LIM domain protein 1, PDLIM1）。有5个未能通过生物信息学方法鉴定。图 3是Xca凝胶图中6号蛋白质点的PMF及Mascot搜索结果。

**2:** Mascot软件搜索数据库鉴定出的蛋白质点 Protein spots identified by Mascot software in database

No.	Description	Accession code	Mw (Da)/pI	Score
1	Superoxide dismutase [Cu-Zn]	P00441	16, 154/5.7	57
2	Triosephosphate isomerase	P60174	26, 938/6.54	57
3	Glutathione S-transferase P	P09211	23, 569/5.34	67
4	ETHE1	O95571	26, 491/6.05	81
5	Alpha enolase	P06733	47, 350/6.99	74
6	Proapototic caspase adapter protein	Q8WU39	21, 023/5.37	78
7	Galectin-3	P17931	31, 888/4.84	80
8	Annexin A1	P04083	38, 918/6.57	92
9	COMT2	Q8WZ04	20, 374/5.56	121
10	Annexin A4	P09525	36, 088/5.84	159
11	PDZ and LIM domain protein	O00151	36, 505/6.56	115
12	Nucleoside diphosphate kinase A	P15531	17, 309/5.83	90
13	Transthyretin	P02766	15, 991/5.52	185
14	Annexin A2	P07355	38, 808/7.57	185
Accession code: Accession codes refer to the Swiss-Prot database.				

**3:**
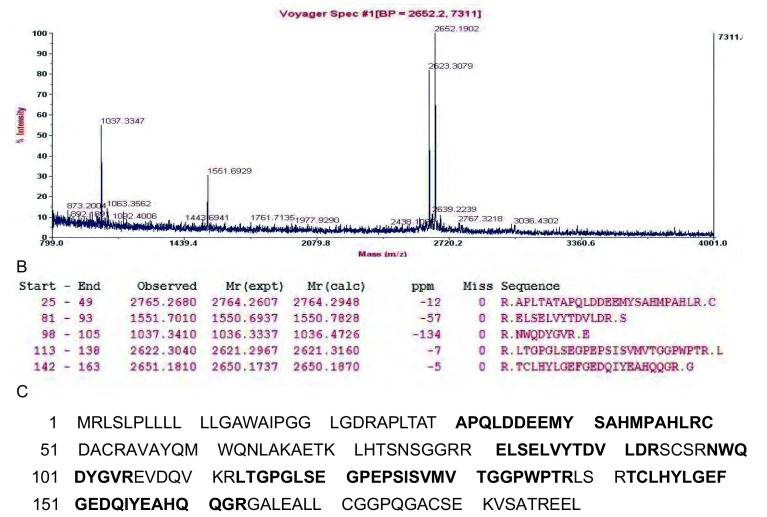
PACAP的PMF及Mascot搜索结果。A：通过MALDI-TOF质谱仪得到的肽指纹图。B：搜索数据库得到的与PACAP相吻合的5个肽段质谱峰。C：PACAP氨基酸序列，粗体字母代表质谱检测到的肽段序列与PACAP的匹配情况。 Identification of PACAP by peptide mass fingerprint. A: Peptide mass spectrum was obtained by MALDI-TOF mass spectrometry; B: The masses of five typical peptides were matched with PACAP by database searching; C: The sequence of PACAP is represented by single-letter code for amino acids. Sequence coverage by five peptides is indicated with bold capital letters.

### 2.3 Western blot检测

第6号蛋白质点是双向电泳中发现的表达差异比较明显的蛋白质，经鉴定是PACAP，已有的研究发现PACAP有一定的组织特异性，在脑组织中高表达，未见其在肿瘤患者血清中的研究。我们选取它作为进一步研究的对象，检测了4组培养液上清及6组血清标本中PACAP的表达情况。结果见图 4、图 5。结果显示PACAP在肺腺癌患者血清中的表达水平是它在健康人血清中的2.51倍-5.81倍，平均（3.56±1.16）倍。

**4:**
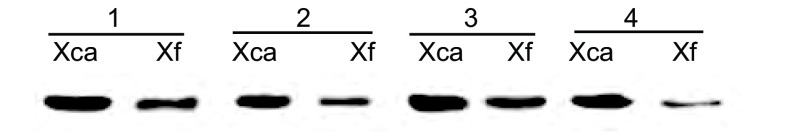
PACAP在4组肺腺癌（Xca）和配对肺组织（Xf）培养液上清中的Western blot结果 Highly expressed PACAP in lung adenocarcinoma tissues conditioned medium. The expression levels of PACAP were compared by immunoblotting in conditioned medium between lung adenocarcinoma (Xca) and paired lung tissue (Xf).

**5:**
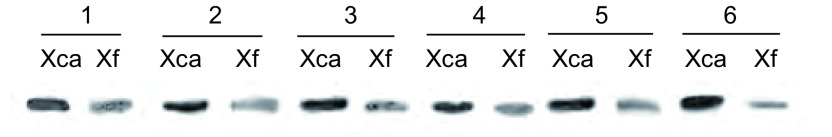
PACAP在6组肺腺癌（Xca）和正常人血清（Xf）标本中的Western blot结果 Highly expressed PACAP in lung adenocarcinoma serum. The expression levels of PACAP were compared by immunoblotting in serum between lung adenocarcinoma (Xca) and health people (Xf).

### 2.4 PACAP ELISA检测

肺腺癌血清PACPA的*OD*值是0.188±0.033，较肺小细胞癌（*OD*值为0.151±0.017）（*P*=0.002）、肺良性病变（*OD*值为0.154±0.023）（*P* < 0.001）和健康人（*OD*值为0.147±0.020）（*P* < 0.001）均高，差异具有统计学意义。肺腺癌和肺鳞癌之间*OD*值差别不明显（*P*=0.704）。肺小细胞癌和肺良性肿瘤（*P*=0.939）以及健康人（*P*=0.346）之间*OD*值差别不明显（表 3）。

**3:** ELISA标本资料及血清中PACAP的ELISA检测结果 Clinical data and results of PACAP by ELISA analysis

Group	Gender (Male/Female)	Age (yr)	*n*	*OD*
Adenocarcinoma	22/23	51+17	45	0.188+0.033
Squamous cell carcinoma	20/23	48+10	43	0.186+0.034
Small cell lung cancer	5/3	45+19	8	0.151+0.017
Lung benign tumor	17/11	41+23	28	0.154+0.023
Healthy control	12/8	48+18	20	0.147+0.020

## 3 讨论

我们选用前期成功建立的基于鼠尾胶原的组织培养模型进行肿瘤组织的培养，收集长势良好的组织的培养液，先过滤坏死的细胞及其它杂质后，再进行二维电泳及蛋白质鉴定，经过PDQuest软件比较匹配，发现19个表达差异的蛋白质，鉴定出了14个，其中9个表达上调，5个表达下调。鉴定的蛋白质PACAP在非小细胞肺癌患者的组织培养液中浓度明显高于对照组，随后我们用Western blot和ELISA检测了血清标本中的PACAP情况，发现它在非小细胞肺癌患者血清中的表达情况明显高于其它对照组，因此，我们认为PACAP可能是非小细胞肺癌潜在的血清标志物。

组织细胞在人体内生长时，会与邻近的细胞和胞外的基质建立复杂的生化和机械联系，形成一个独特的组织微环境。这个微环境就是普通的平面培养和三维培养的最大区别之所在。正是它的存在使三维培养可以更加真实地模拟组织细胞在体内的生长情况。目前很多来自细胞生物学、生物化学以及药理学等学科的研究发现，平面培养模式下的结果往往与真实情况存在差异，而且也越来越难以满足新的研究要求，而以三维培养为平台在耐药性^[8]^、基因表达^[9]^等方面的研究结果已经与临床情况非常接近。在前期的研究中，我们经过反复对比和挑选，最后成功建立了基于鼠尾胶原的组织培养平台——一种易行的三维培养方式^[6]^。我们希望通过这个平台，能在肺癌血清标志物的筛选上有新的发现。

蛋白质组学技术是分离和鉴别大量蛋白质的强有力工具，我们利用蛋白质组学技术分析3D培养条件下的培养液上清，鉴定出了14个差异表达的蛋白质。它们大体上可以分为如下几类：代谢相关蛋白，如NNE、Tim、NDPKA、COMT2；信号传导蛋白，如ANXA1、ANXA2、ANXA4、Galectin-3、PACAP；抗氧化蛋白，如SOD1、GSTP1；转运蛋白，如TTR、ETHE1；结构蛋白，如PDLIM1。这些蛋白中，NNE、Tim、NDPKA、ANXA家族、Galectin-3、SOD-1及GSTP-1在以往的研究中已发现与肺癌的发生、发展及转移有关系。

PACAP是本研究中发现的表达上调差异比较明显的蛋白质，也未见到有相关文献报道其与肺癌的关系，因此，我们选取它作为研究对象。PACAP作为一种凋亡调控相关蛋白，编码基因位于5q23-q31，长度2, 411 bp。它由189个氨基酸构成，分子量为20, 694 Da，通过与Caspase-2和Caspase-9结合参与凋亡调控。它在细胞中定位于胞浆，呈弥漫性地颗粒样分布于核周。PACAP有一定的组织特异性，在人脑组织中高表达，在其它组织中表达相对较低^[10]^。它有3种同分异构体，但只有一种具有完整的189个氨基酸序列。与很多凋亡通路成员不同的是，PACAP并不包含CARD（caspase recruitment domain）——这是很多Caspase和凋亡信号蛋白相互作用的结构。它包含的是一个名为WD40的氨基酸末端，这个末端的22个氨基酸形成一个P型的袢结构，发挥着信号肽的作用^[10]^。迄今为止，针对PACAP的研究极少，本研究使用差异蛋白质组学技术首次发现它在肺腺癌组织培养液上清中高表达，并采用ELISA检测了它在不同人群共144例血清标本中的表达情况，发现它在肺腺癌和肺鳞癌患者血清中高表达，与它在小细胞肺癌患者、肺部良性疾病患者和健康人血清中的水平差异明显。血清中的PACAP水平无法区别肺腺癌和肺鳞癌。在区分小细胞肺癌和肺良性肿瘤时，也没有明显的意义。一方面可能是本研究的小细胞肺癌样本量少，无法代表群体情况，使结果出现偏倚，另一方面可能是由于小细胞肺癌的生物学特异性，分泌的PACAP确实不存在差异。

在采用蛋白质组学研究的过程中，经常出现找到的差异蛋白质点表达情况与随后验证结果不同的情况。因此，我们先直接选取培养液上清和人血清为研究对象，采用Western blot检测来校正蛋白质组学的实验结果。由于培养液上清和人血清样品不同于细胞总蛋白样品，没有β-actin、β-tubulin等可供对比的内参。研究只能在保证上样量一致的情况下，粗略比较目标蛋白的差异。这是国际上直接比较分泌蛋白和上清蛋白常用做法^[11-13]^。ELISA是一种定量检测方法，在设置严格对照的情况下，得到的结果特异性好，灵敏度高，而且试验流程较简单，适合较大规模筛查。因此研究选择ELISA进行例数较多的血清检测。

本研究发现PACAP是非小细胞肺癌的潜在血清标志物。为了进一步明确PACAP在非小细胞肺癌诊断中的作用，后续还需要进一步研究PACAP与非小细胞肺癌分期的关系，更大样本量检测小细胞肺癌患者血清中PACAP的表达情况，对人群进行前瞻性的研究确定PACAP在诊断早期非小细胞肺癌上的效能，将PACAP与其它血清标志物联合检测争取发现敏感性和特异性更好的组合，PACAP与非小细胞肺癌预后和复发的关系?有关PACAP的生物学特性、PACAP为什么在非小细胞肺癌中高表达以及它在非小细胞肺癌的发生发展中起到什么样的作用也需要进一步的研究。

## References

[1] Jemal A, Siegel R, Ward E (2008). Cancer statistics, 2008. CA Cancer J Clin.

[2] Humphrey LL, Teutsch S, Johnson M (2004). Lung cancer screening with sputum cytologic examination, chest radiography, and computed tomography: an update for the U.S. Preventive Services Task Force. Ann Intern Med.

[3] Diederich S (2008). CT screening for lung cancer. Cancer Imaging.

[4] Huang LJ, Chen SX, Huang Y (2006). Proteomics-based identification of secreted protein dihydrodiol dehydrogenase as a novel serum markers of non-small cell lung cancer. Lung Cancer.

[5] Huang LJ, Chen SX, Luo WJ (2006). Proteomic analysis of secreted proteins of non-small cell lung cancer. Ai Zheng.

[6] Zhang H, Chen SX, Zhang CF (2008). Three-dimensional culture of the primary lung adenocarcinoma tissues. Zhong Liu.

[7] Gorg A, Obermaier C, Boguth G (2000). The current state of two-dimensional electrophoresis with immobilized pH gradients. Electrophoresis.

[8] David L, Dulong V, Le Cerf D (2008). Hyaluronan hydrogel: an appropriate three-dimensional model for evaluation of anticancer drug sensitivity. Acta Biomater.

[9] Becker JL, Blanchard DK (2007). Characterization of primary breast carcinomas grown in three-dimensional cultures. J Surg Res.

[10] Bonfoco E, Li E, Kolbinger F (2001). Characterization of a novel proapoptotic caspase-2- and caspase-9-binding protein. J Biol Chem.

[11] Oceguera LF 3rd, Patiris PJ, Chiles RE (2007). Flavivirus serology by Western blot analysis. Am J Trop Med Hyg.

[12] Reid DM, Jones CE, Vostal JG (1990). Western blot identification of platelet proteins that bind normal serum immunoglobulins. Characteristics of a 95-Kd reactive protein. Blood.

[13] Dai DF, Thajeb P, Tu CF (2008). Plasma concentration of SCUBE1, a novel platelet protein, is elevated in patients with acute coronary syndrome and ischemic stroke. J Am Coll Cardiol.

